# Vitamin D and Immune Response: Implications for Prostate Cancer in African Americans

**DOI:** 10.3389/fimmu.2016.00053

**Published:** 2016-02-22

**Authors:** Ken Batai, Adam B. Murphy, Larisa Nonn, Rick A. Kittles

**Affiliations:** ^1^Division of Urology, Department of Surgery, The University of Arizona, Tucson, AZ, USA; ^2^Department of Urology, Feinberg School of Medicine, Northwestern University, Chicago, IL, USA; ^3^Jesse Brown Veterans Affairs Medical Center, Chicago, IL, USA; ^4^Department of Pathology, University of Illinois at Chicago, Chicago, IL, USA

**Keywords:** vitamin D, prostate cancer, inflammation, African Americans, health disparities

## Abstract

Prostate cancer (PCa) is the most common cancer among men in the U.S. African American (AA) men have a higher incidence and mortality rate compared to European American (EA) men, but the cause of PCa disparities is still unclear. Epidemiologic studies have shown that vitamin D deficiency is associated with advanced stage and higher tumor grade and mortality, while its association with overall PCa risk is inconsistent. Vitamin D deficiency is also more common in AAs than EAs, and the difference in serum vitamin D levels may help explain the PCa disparities. However, the role of vitamin D in aggressive PCa in AAs is not well explored. Studies demonstrated that the active form of vitamin D, 1,25-dihydroxyvitamin D, has anti-inflammatory effects by mediating immune-related gene expression in prostate tissue. Inflammation also plays an important role in PCa pathogenesis and progression, and expression of immune-related genes in PCa tissues differs significantly between AAs and EAs. Unfortunately, the evidence linking vitamin D and immune response in relation to PCa is still scarce. This relationship should be further explored at a genomic level in AA populations that are at high risk for vitamin D deficiency and fatal PCa.

## Introduction

Prostate cancer (PCa) is the most common cancer among men in the U.S., and African American (AA) men have higher PCa incidence and mortality than other racial/ethnic groups. Social and behavioral factors affect stage, grade, treatment choice, and mortality (1, 2), but the etiologies for the PCa disparities are still being elucidated. Epidemiologic studies have showed that vitamin D deficiency is associated with advanced stage, higher tumor grade, and mortality (3–5), while its association with overall PCa incidence is inconsistent (6–9). Vitamin D deficiency is also common in AAs, even AAs living in southern U.S. (10–12), and differences in serum vitamin D levels may help explain PCa disparities (13, 14). However, epidemiologic studies have mainly been conducted in men of European descent, and the role of vitamin D in AAs, who are more likely to have aggressive PCa, has not been well explored.

Several pathways for how vitamin D affects PCa pathogenesis and progression have been explored. The active form of vitamin D, 1,25-dihydroxyvitamin D [1,25(OH)_2_D], inhibits tumor cell proliferation and induces apoptosis (15, 16) 1,25(OH)_2_D also modulates expression of immune-related genes in prostate tissue (17). 1,25(OH)_2_D binds to vitamin D receptor (*VDR*) and regulates expression of hundreds of genes that have vitamin D response elements (VDREs), a segment of DNA found in the promoter region of vitamin D target genes. This transcriptional regulation by 1,25(OH)_2_D and VDR can affect the production of immune-related biomarkers (18, 19). Inflammation also plays an important role in PCa pathogenesis (20), and expression of immune-related genes in PCa tissues differs significantly between AAs and European Americans (EAs) (21–23).

In this review, we discuss the relationships and interactions between vitamin D and immune response. Many recent studies have shown the role of vitamin D on immune response, but the evidence linking vitamin D and immune response in the context of PCa is still scarce. We argue that this relationship should be investigated at genomic level, especially in AA populations that are at high risk for both PCa and vitamin D deficiency.

## Prostate Cancer Disparities and African Americans

African American men have a 59% higher incidence and more than a twofold higher mortality rate compared to EA men (24). AAs are often diagnosed with PCa at younger ages and have PCa with a higher Gleason score, clinical stage, and prostate-specific antigen (PSA) level (25–28). AA patients with low risk PCa tend to have worse clinical features after undergoing prostatectomy (29, 30). Molecular differences in tumors from AAs and EAs exist and may result in a faster growth or earlier transformation to aggressive PCa in AAs compared to EAs (31–33). The cause of tumor biological differences between AAs and EAs is still unknown, but ancestry-related factors, such as genetic variation, vitamin D deficiency, and immune functions, may contribute.

## Vitamin D and Prostate Cancer

Vitamin D is believed to have protective effects on PCa, especially for aggressive PCa (34, 35). Epidemiologic studies have shown that PCa patients with low serum 25-hydroxyvitamin D [25(OH)D] levels are likely to have higher PCa stage, grade, and mortality (3, 4, 36–38). On the other hand, association with overall PCa risk is inconsistent, and many studies have shown no association (4, 6, 7, 9, 39–42). Interestingly, the Selenium and Vitamin E Cancer Prevention Trial revealed that both low and high vitamin D levels increased PCa risk (43). Because vitamin D deficiency is very common among AAs (44–49), the higher prevalence of vitamin D deficiency may account for a proportion of PCa disparities (13, 14, 34). However, only a few studies demonstrated the association of vitamin D and PCa in AAs (5, 50).

**Figure 1:**
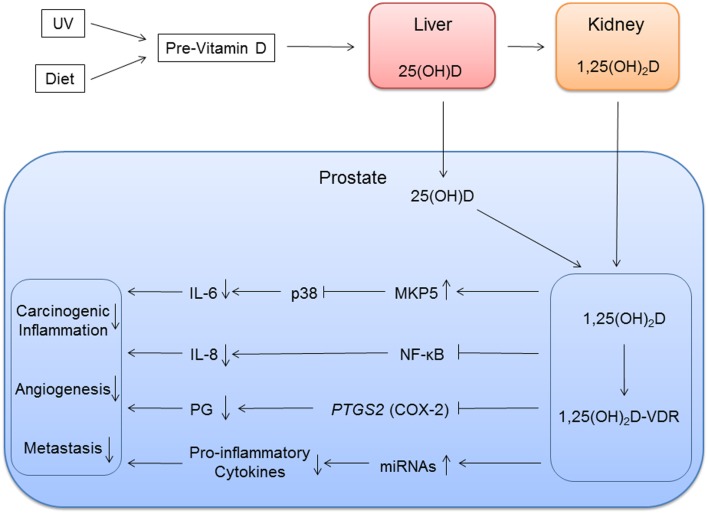
**Vitamin D inflammation pathways of prostate cancer**. Vitamin D metabolites are transported to the prostate. 25(OH)D is locally metabolized to 1,25(OH)_2_D, and 1,25(OH)_2_D binds to vitamin D receptor (VDR) with coactivators. This vitamin D and VDR complex modulates immune response by regulating expression of vitamin D target genes. The genes involved in immune response can be direct targets with vitamin D response element or in downstream in the pathways.

25(OH)D is the main circulating form of vitamin D, and it is metabolized to the biologically more active, but less abundant, 1,25(OH)_2_D in the kidney by 1α-hydroxylase (Figure 1) (51). Both forms of vitamin D are transported to the prostate and other organs, and 1α-hydroxylase present in the prostate converts 25(OH)D to 1,25(OH)_2_D (52). Because 25(OH)D is more abundant than 1,25(OH)_2_D, both metabolites may have important roles in the prostate. Results of various experiments suggest that 1,25(OH)_2_D inhibits growth of prostate epithelial cells and PCa cells by inducing cell cycle arrest and apoptosis (15, 53–55). Vitamin D inhibits the tumor cell proliferation and induces apoptosis through activities of the *VDR* (56). 1,25(OH)_2_D attaches to the VDR, a transcription factor that binds to VDREs usually in the promoter region of vitamin D-responsive genes. Subsequently, activated VDR interacts with coactivators or corepressors to activate or repress these vitamin D-responsive genes. The *VDR* is expressed in prostate epithelium (57). Expression of *VDR* decreases after age 60 (58), and PCa patients with low *VDR* expression are more likely to have advanced and lethal PCa (59).

## Inflammation and Prostate Cancer

Inflammation may play a role in PCa pathogenesis and progression (20, 60–62), but epidemiologic studies have shown conflicting evidence. In the placebo arm of the Prostate Cancer Prevention Trial, men who had at least one biopsy core with inflammation in benign prostate tissue had increased odds of overall PCa and high grade PCa (63). In the REduction by DUtasteride of Prostate Cancer Events (REDUCE), use of aspirin and/or non-steroid anti-inflammatory drugs (NSAIDs) reduced odds of overall PCa and high-grade PCa (64), but baseline acute and chronic inflammation was associated with reduced PCa risk at follow-up (65).

Although evidence linking inflammation and PCa is limited in EA populations, and prostate biopsy specimens from AA patients revealed infiltration of immune cells more frequently than specimens from EA patients (66). The difference in the inflammatory prostatic microenvironment between AAs and EAs may explain some of the PCa disparities. Microarray gene expression studies revealed different gene expression patterns between AA and EA prostate tumors for genes in immune-related pathways, including cytokines (e.g., *IL1B*, *IL6*, and *IL8*), which were over-expressed in PCa tissues from AAs compared to tissues from EAs (21–23, 67). Many genes were also differentially expressed in the stromal compartment, and approximately 20% of the identified pathways are immune-related, especially cytokine-mediated pathways (23). Giangreco et al. (57) also found that expression of *IL6* is more than 18-fold higher in PCa-associated stroma than in PCa or benign epithelium. These studies suggested that both stromal and epithelial cells differentially express immune-related genes and contribute to the inflammatory environment. The stroma surrounding epithelium is known to play an important role in prostate development as well as PCa progression. The stroma microenvironment is complex and consists of monocytes, macrophages, T cells, and neutrophils alongside smooth muscle cells, myofibroblasts, fibroblasts, and collagen fibers (68). Stromal components regulate epithelial cell differentiation and proliferation and also mediate immune response of epithelial cells. The prostatic stroma becomes reactive early in PCa development and coevolves with epithelial cells during progression (68, 69).

Genetic variants may affect expression of genes involved in immune response and also angiogenesis. Genetic studies conducted mainly in EAs showed associations of inflammation pathway gene single nucleotide polymorphisms (SNPs) with PCa, and evidence of associations with aggressive PCa was stronger (70–74). Recent studies in African descent populations also showed that SNPs in many inflammatory genes were associated with PCa (74–79). Allele frequencies of cytokine gene SNPs differ significantly between AAs and EAs, and the frequencies of alleles that upregulate proinflammatory cytokines are higher in AAs than in EAs (80, 81). Cytokines modulate immune response involved in angiogenesis, and proinflammatory cytokine levels are elevated in advanced PCa patients (82–84). The levels of inflammatory markers in serum also vary between racial/ethnic groups, and AAs have higher levels of circulating proinflammatory markers than EAs (85, 86).

However, the effects of genomic variants on the expression of inflammatory genes and the production of cytokines have not been well explored. Moreover, chronic inflammation may cause epigenetic changes and genomic instability, which may promote aggressive PCa in AAs. Additionally, more research is needed to uncover biologically significant environmental exposures that contribute to the differential immune response between AAs and EAs.

## Vitamin D and Inflammation

In addition to calcium homeostasis, vitamin D also functions as a modulator of innate and adaptive immune response. Population-based studies, as well as molecular studies, have demonstrated that vitamin D is implicated in many immune-related diseases, such as asthma, atherosclerosis, type 2 diabetes, and autoimmune diseases (87, 88). Serum 25(OH)D levels are also inversely associated with circulating proinflammatory cytokine levels (89–96). Vitamin D supplementation and fortification likely reduce serum proinflammatory markers’ levels (97–101), but other supplementation trials have shown no significant effects of vitamin D supplementation on proinflammatory markers levels (102–105). One study explored the relationship between circulating 25(OH)D and proinflammatory markers in AAs (105). In that study, baseline 25(OH)D levels was significantly associated with C-reactive protein levels, but 3 months of supplementation did not affect inflammatory markers’ levels. These studies varied in the participants’ baseline 25(OH)D levels, length of the trials, and supplementation dosage. In addition, individual genetic variation in vitamin D metabolism and signaling may impact response to vitamin D supplementation and effectiveness of supplementation to regulate inflammatory response (106).

Vitamin D modulates immune response by regulating expression of immune-related genes, such as cytokines, in very complex ways through the VDR activities. VDR is a nuclear transcription factor that interacts with a multitude of signaling pathways and thereby regulates the inflammatory response through transcription (88). Genome-wide screening using non-prostate cell lines (immune cells and colorectal cancer cells) recently identified over 10,000 new VDR-binding sites (107), and many cytokines, cytokine receptors, and other immune-related genes were identified as VDR targets (108–110). Vitamin D supplementation can also alter the expression of genes involved in immune response, including *CD14*, the gene that encodes surface antigen expressed on monocytes and macrophages, and *NFKBIA*, a gene for a protein that inhibits NF-κB, which plays a key role in regulating the immune response to infection (106, 111). Other studies found that polymorphisms in the *VDR* affect cytokine expression and protein production in peripheral blood mononuclear cells (112–114).

1,25(OH)_2_D binds to the VDR present on B cells, T cells, and antigen-presenting cells and affects the local immunologic milieu (115). The *VDR* gene expression and VDR signaling affect T cell development, differentiation, and function (116). *In vitro* studies have also shown that 1,25(OH)_2_D reduces production of proinflammatory cytokines, including IL-6, IL-8, and tumor necrosis factor α (TNF-α), in monocytes, macrophages, and preadipocytes (117–119). The prostate, like many other organs, harbors immune cells, and the cytokines produced by immune cells in the prostate may promote PCa pathogenesis, proliferation, and metastasis.

## Implications for Prostate Cancer

In the prostate, 1,25(OH)_2_D inhibits production of proinflammatory molecules that contribute to PCa initiation and growth (16, 17), but the molecular pathways involving vitamin D and inflammation in the context of PCa is not well explored. We know that for prostate epithelial cells treated with 1,25(OH)_2_D, *TNF*α, *IL6*, and *IL8* expression is suppressed, while *TNF*α and *PTGS2* expression is suppressed in stromal cells (57). *PTGS2* encodes COX-2, cyclooxygenase 2, an enzyme that converts arachidonic acid to proinflammatory prostaglandins. *PTGS2* levels are higher in PCa (120, 121) and 1,25(OH)_2_D suppressed *PTGS2* expression in PCa cells (122). 1,25(OH)_2_D also inhibits NF-κB signaling reducing IL-8 production (123). NF-κB is a protein complex involved in the regulation of transcription of numerous genes involved in inflammatory and immune response. Specifically, 1,25(OH)D reduces downstream production of IL-8 production by inhibiting the binding of NF-κB to DNA response elements (Figure 1).

Genome-wide screening using non-prostate cell lines found little overlap in VDR-binding sites, suggesting VDR binding is cell-specific (107). Thus, a study using prostate tissue is necessary in order to identify prostate-specific VDR-binding sites and to further understand the role of vitamin D in PCa. However, microarray studies using PCa cell lines have identified some *VDR* targets, and some of the targets are genes mediating downstream productions of cytokines (18, 19). One of the VDREs that were identified is mitogen-activated protein kinase phosphates 5 (MKP5). MKP5 was upregulated in response to 1,25(OH)_2_D treatment (18). Upregulation of MKP5 inactivated p38 resulting in reduced IL-6 production (124). 1,25(OH)_2_D also attenuated TNF-α-stimulated p38 activity to reduce IL-6 production.

1,25(OH)_2_D can also impact inflammation and PCa through its regulation of microRNAs (miRNAs) expression. miRNA is a small non-coding RNA molecule of about 22 nucleotides that has post-transcription gene regulatory functions. Studies have identified miRNAs that are regulated by 1,25(OH)_2_D (125–128), and eight miRNAs, including miR-100 and mi125b, were positively correlated to prostatic 1,25(OH)D levels from PCa tissues from the vitamin D supplementation clinical trials (129, 130). Some of these miRNAs are also involved in the regulation of cancer-associated inflammatory response (16, 131).

The aforementioned studies provided some mechanistic insights into vitamin D regulation of prostatic inflammation. PCa develops and grows slowly over decades, indicating that the protective effects of vitamin D must include regulatory processes other than cell proliferation. Given the well-characterized actions of vitamin D on immune cells, vitamin D’s anti-inflammatory actions are likely to influence PCa initiation and progression. However, previous studies have only identified a few vitamin D inflammatory pathways, which could putatively lead to PCa, and there are other relationships between vitamin D and immune-related genes for PCa initiation and progression remain unexplored. Further investigation is necessary to elucidate the mechanisms, by which vitamin D regulates immune gene expression in indolent and aggressive prostate tumors.

## Clinical Significance and Future Directions

Understanding how vitamin D affects PCa initiation and progression may contribute to the development of better primary prevention and therapeutic strategies using vitamin D supplementation, especially AAs who are at high risk for both vitamin D deficiency and aggressive PCa. In a vitamin D supplementation trial among healthy AA men from Boston, 3 months of supplementation use did not lower PSA levels (132), but there are several clinical trials that have demonstrated benefits of vitamin D supplementation in PCa patients. PSA velocity and PSA doubling time are strong predictors of PCa mortality, and persistently rising PSA levels after radical prostatectomy or radiation therapy indicates biochemical recurrence (133). In vitamin D supplementation trials among PCa patients, supplementation reduced PSA levels and rate of PSA rise, and increased PSA doubling time (130, 134, 135). In a pilot clinical trial of low risk PCa patients who were on active surveillance and had a repeat biopsy at 1 year (27% AAs), supplementation did not lower their PSA levels, but men on supplements had a decreased number of positive cores and no increase in Gleason Score (136). In another vitamin D supplementation trial in Canada that included four (6%) black Canadians, prostatic 25(OH)D and 1,25(OH)_2_D were significantly higher in supplement groups (130). In laser-capture microdissected PCa epithelium from the study, *PTGS2* expression was lower in the highest prostatic 1,25(OH)_2_D tertile compared to the lowest tertile (57).

In these vitamin D supplementation trials, supplementation improved clinical characteristics of many PCa patients but not all. Future studies need to investigate dosage necessary for the non-responders and genetic variations, epigenomic changes, and biological and behavioral factors that modify the efficacy of vitamin D supplementation in non-responders. For example, *VDR* and inflammatory gene variants may alter the effectiveness of vitamin D supplementation. It is also possible that combined use of vitamin D supplement and NSAIDs that inhibit COX-2 enzymatic activities is more effective for primary prevention and clinical management (17, 122).

Despite the high PCa incidence and mortality in AAs and tumor biological differences between AAs and EAs, AAs are still underrepresented in PCa epidemiologic studies, clinical trials, and molecular mechanistic studies. Differences in genetic variation partly account for the PCa disparities between AAs and other racial/ethnic groups (137–141). However, genome-wide association study in AAs did not identify immune and vitamin D-related gene variant as a risk locus for PCa, and it is likely that behavioral and biological factors, such as serum vitamin D levels, modify the associations between immune and vitamin D-related gene variants and PCa. Future studies need to explore these behavioral and biological factors that modify the relationship between PCa and immune response. Vitamin D may modify associations between sequence variants in immune-related genes and PCa. Sequence variants, especially in and around VDRE, may have heterogeneous effects on PCa between vitamin D-deficient and -sufficient individuals.

Future studies also need to explore the epigenomic effects of vitamin D. Vitamin D may regulate immune response through epigenetic mechanisms. Vitamin D supplementation may induce epigenetic changes to VDR and VDR targets (142, 143). Diverse methylation patterns were observed between a 1,25(OH)_2_D-responsive non-malignant prostate cell line and a non-responsive PCa cell line after treatment with vitamin D (144). It is clear though those actions of the VDR are very complex. Epigenetic regulations by VDR involve interactions with corepressors, such as NCOR1, histone deacetylases (HDACs), and miRNA, to repress transcription and with coactivators and histone acetyltransferases (HATs) for gene transcription (142, 145). However, the epigenetic regulatory role of vitamin D in inflammatory response in PCa cells has not been explored.

In conclusion, chronic vitamin D deficiency may create a tumor microenvironment with increased inflammation. This type of tumor microenvironment could be more common in PCa from AA patients than EA patients or could cause tumors to become more aggressive. However, the vitamin D inflammation pathways have not been a well-explored mechanism in PCa pathogenesis and progression. Future studies need to explore this relationship in order to improve our understanding of the biologic basis of PCa health disparities.

## Author Contributions

KB, ABM, LN, and RAK each contributed to the literature review and drafting of this manuscript.

## Conflict of Interest Statement

The authors declare that the research was ­conducted in the absence of any commercial or financial relationships that could be construed as a potential conflict of interest.
